# Hatched Eggshell Membrane Can Be a Novel Source of Antioxidant Hydrolysates to Protect against H_2_O_2_-Induced Oxidative Stress in Human Chondrocytes

**DOI:** 10.3390/antiox11122428

**Published:** 2022-12-09

**Authors:** Lingjiao Zhu, Meihu Ma, Dong Uk Ahn, Vincent Guyonnet, Limei Wang, Yuting Zheng, Qin He, Hanguo Xiong, Xi Huang

**Affiliations:** 1 College of Food Science and Technology, Huazhong Agricultural University, Wuhan 430070, China; 2 National R&D Branch Center for Egg Processing, Jingmen 431800, China; 3 Animal Science Department, Iowa State University, Ames, IO 50011, USA; 4 FFI Consulting, Limited, 2488 Lyn Road, Brockville, ON K6V 5T3, Canada

**Keywords:** oxidative stress, chondrocytes, antioxidant hydrolysates, hatched eggshell membrane, Keap1/Nrf2/HO-1 pathway

## Abstract

Natural antioxidants derived from agricultural by-products have great promise and ecological advantages in the treatment of oxidative stress-related diseases. The eggshell membrane (ESM) from hatched eggs, i.e., the hatched ESM, is a globally abundant agricultural byproduct, and its high-value utilization has been rarely studied compared to the well-studied ESM from fresh eggs. In this research, we systematically characterized the hatched ESM as a novel source of antioxidant hydrolysates and explored their potential role in H_2_O_2_-induced human chondrocytes. The results showed that the hatched ESM is a protein-rich fibrous mesh material with a significantly different structure and composition from those of fresh ESM. Enzymatic hydrolysis of hatched ESM can produce antioxidant hydrolysates rich in low molecular weight (MW) peptides, which mainly derived from the Lysyl oxidase homolog by Nano-LC-MS/MS analysis. The peptide fraction with MW < 3 kDa (HEMH-I) exhibited the highest DPPH radical scavenging, Fe^2+^-chelating, and Fe^3+^-reducing abilities. In H_2_O_2_-induced human SW1353 chondrocytes, HEMH-I treatment significantly increased the cell viability and ameliorated oxidative stress, inflammatory response, and cartilage matrix degradation by reducing the level of ROS, matrix metalloprotease 3 (MMP3), MMP13, and IL-6, and by promoting the expression of SOD and type II collagen, potentially through activating the cellular Keap1/Nrf2/HO-1 pathway. This study provides a theoretical basis for the value-added application of hatched ESM waste to produce antioxidant hydrolysates and indicates their potential as functional food and pharmaceuticals.

## 1. Introduction

Oxidative stress occurs when the production of reactive oxygen species (ROS) in the body is excessive and overwhelms the defense capacity of living cells, leading to tissue damage and various chronic diseases, including osteoarthritis (OA), cardiovascular disease, and cancer [1,2]. Among them, OA is a degenerative joint disease induced by oxidative stress in chondrocytes [3,4] and has been considered a major public health concern worldwide [5]. Oxidative stress generated by the accumulation of ROS in chondrocytes can trigger impaired cartilage extracellular matrix (ECM) metabolism and overproduction of proteolytic enzymes and inflammatory cytokines, thereby accelerating cartilage degeneration [4,6]. Therefore, the suppression of oxidative stress injury to chondrocytes is proposed as a potential treatment strategy in OA [3,7]. Due to their high bioactivity and superior safety profile over synthetic antioxidants and drugs, antioxidant hydrolysates or peptides hold great promise for alleviating oxidative stress [8,9]. In recent years, many protein hydrolysates derived from agriculture byproducts, such as chicken cartilage hydrolysates [9], collagen hydrolysates from pork skin, and bovine bone [10], are reported to have antioxidant activity and shown the potential to preventing oxidative stress-related disease.

Globally, the amount of eggshell and eggshell membrane (ESM), by-products generated annually by the poultry and egg-processing industry, is estimated at >7 million tons each year and this waste material is ranked 15th in the list of major food industry pollution problems [11,12]. Since ESM waste is a natural biomaterial with high protein content (~90%) [13], producing bioactive protein hydrolysates and peptides from ESM will not only create added value but also significantly improve the environmental sustainability of the poultry and egg industry [13,14]. ESM from fresh eggs (i.e., fresh ESM) in egg-processing plants has been well studied and demonstrated to produce hydrolysates with many bioactivities, including antioxidant and anti-osteoarthritic effects [15,16,17]. However, the ESM from post-hatched eggs, i.e., hatched ESM, as another main source of waste in the poultry industry [18,19,20] has been rarely studied. It is well established that the structure and composition of protein precursors have a critical influence on the release and activity of bioactive peptides in hydrolysates. Based on the biochemical effects of the incubation process on ESM, the structure and composition of incubated ESM may differ significantly from those of fresh ESM [21], as evidenced by previous proteomics analyses of the two ESMs [22,23]. This may result in a difference between the chemical composition and biological activities in the hydrolysates derived from the two ESMs. Our recent work firstly revealed the potential of hatched ESM to prepare antioxidant peptides [24], which needed further investigation to develop this agricultural waste as a novel source of bioactives. In addition, it is worth investigating whether the bioactive hydrolysates derived from the hatched ESM have similar functional activities to that of fresh ESM in ameliorating OA progression. Accordingly, an in-depth research on the characterization of hatched ESM and the biological activity of their derived protein hydrolysates may have important implications for recycling and reusing this agricultural waste material.

Therefore, the aims of this study are (a) to characterize the hatched ESM as a novel source of antioxidant hydrolysates, using the well-studied fresh ESM as control; and (b) to investigate the potential effects and underlying mechanism of hatched ESM hydrolysates in H_2_O_2_-induced human SW1353 chondrocytes. In particular, their role in regulating the Keap1/Nrf2/HO-1 pathway, a major pathway in protecting cells from oxidative stress [25] and a novel therapeutic target for OA [6], was taken into account. The strategy of this work is shown in Figure S1. This study provides insights into the characteristics of hatched ESM to prepare antioxidant hydrolysates and a theoretical basis for their high-value-added utilization in functional food and in the pharmaceutical industry. 

## 2. Materials and Methods

### 2.1. Materials and Chemicals

Fresh eggshells (i.e., eggshells from fresh unfertilized chicken eggs) and hatched eggshells (i.e., eggshells from post-hatch chicken eggs) were obtained from Hubei Shendi Biotechnology Co., Ltd. (Jingmen, China). Membranes from these two sources were cleaned and peeled off the shells according to our previous report [24] and stored at −20 °C until further use. Alcalase was obtained from Novozymes Co., Ltd. (Tianjin, China). Other reagents were bought from Sinopharm Chemical Reagent Co., Ltd. (Shanghai, China). The reagents used in this investigation were analytical grade.

### 2.2. Scanning Electron Microscopy

After hand-peeling, the ESM were cleaned and vacuum freeze-dried, then cut into pieces of about 0.5 × 0.5 cm and coated with gold. The morphology of fresh and hatched ESM was observed using a scanning electron microscope (JSM-6390LV, JEOL, Japan) and images of ESM were obtained at 500×, 1500×, and 3500× magnification.

### 2.3. The Chemical Characterization of ESM

The moisture, ash, and protein content of the ESM from two sources were determined using the AOAC methods [26]. The total sugar content was determined following Jia et al. [27] based on the phenol–sulfuric acid method with glucose as a standard. 

### 2.4. Amino Acid Analysis

The amino acid composition of ESM was analyzed following the method of Jain and Anal [14] with some modifications: 40 mg ESM was added with 6 M HCl before hydrolysis at 110 °C for 24 h. HCl remaining in the hydrolysates was evaporated in a water bath at 70 °C. After re-dissolving and filtering the samples through a 0.22 μm membrane filter, they were analyzed using an amino acid analyzer (MembraPure, A300-advanced, Hennigsdorf, Germany). The amino acid composition was expressed as g/100 g of sample.

### 2.5. Preparation of Eggshell Membrane Protein Hydrolysates

Fresh eggshell membrane hydrolysate (FEMH) and hatched eggshell membrane hydrolysate (HEMH) were prepared according to our previous study. The ESM powder was mixed with alcalase (5%, *w/w*) and distilled water. The pH of the mixture was adjusted to 8.0 before placing the mixture in a 55 °C water bath for 2–14 h. The above reaction was interrupted by heating at 80 °C for 20 min. The pH of the mixture was then adjusted to 7.0. After centrifugation at 8000 rpm for 15 min, the supernatant was dried using a vacuum freeze-dryer and stored at −20 °C.

### 2.6. Degree of Hydrolysis (DH)

The DH of ESM hydrolysates was determined using a pH-stat method as previously reported by Liu et al. [28] using the following equation:

$$\mathrm{DH}\;=\frac{V\left(\mathrm{NaOH}\right)\times N\left(\mathrm{NaOH}\right)}{\alpha \left({\mathrm{NH}}_{2}\right)\times m\left(\mathrm{protein}\right)\times h_{\mathrm{tot}}}$$

where α is the average degree of dissociation of the α-NH_2_ groups, and h_tot_ is the total number of peptide bonds per gram of protein with 7.84 mmol/g and 7.87 mmol/g for fresh and hatched ESM, respectively, calculated based on their amino acid composition.

### 2.7. Determination of Molecular Weight (MW) Distribution

Referring to the method reported by Jia et al. [27], the MW distribution was measured using a high-performance liquid chromatography (HPLC) system (Agilent, Santa Clara County, CA, USA) equipped with a TSK gel G2000 column (300 mm × 7.8 mm; Tosoh, Tokyo, Japan). A total of 10 µL of the sample was separated at a flow rate of 0.5 mL min-1 at 30 °C and monitored at 220 nm. Bovine insulin (5733.49 Da), Bacitracin (1422 Da), Gly-Gly-Tyr-Arg (451 Da), and Gly-Gly-Gly (189 Da) were used to plot the standard curve. The data were analyzed using Breeze software (Waters, MA, USA). The peaks of each sample were integrated and divided into 4 ranges (MW > 5000, 3000–5000, 1000–3000, and <1000 Da). The relative content of each molecular weight range was expressed as the corresponding peak area for each region. The logarithm of the relative molecular mass (log MW) was linearly related to the retention time (Rt) with the following equation: Rt = −0.311558(log MW) + 8.51004 (R^2^ = 0.9867, *p* < 0.05).

### 2.8. Nano-LC-ESI-MS/MS Analysis

The major peptides in the HEMH were sequenced by nano-LC-ESI-MS/MS using an Easy-nLC HPLC system with a reversed-phase C18-A2 column (75 μm × 10 cm, Thermo scientific EASY column,). The loaded samples were eluted with a linear gradient at a flow rate of 300 nL/min using 0.1% formic acid aqueous solution (A) and 0.1% formic acid 84% acetonitrile (B) as mobile phases. Linear gradient: from 0% to 35% B for 50 min, from 35% to 100% B for 5 min, and 100% B for 5 min, were adopted. MS scans were obtained from 300 *m/z* to 1800 *m/z* at 70,000 resolution. A search of the UniProt *Gallus gallus* (chicken) database for data files was performed using MaxQuant (version 1.5.1.2)

### 2.9. Determinati on of Chemical Antioxidative Activity

#### 2.9.1. DPPH Radical Scavenging Activity Assay

The scavenging activity of DPPH radicals was determined using the method of Zhang et al. [29] with some modifications. A total of 100 µL of the sample solution and 100 µL of ethanol DPPH (0.2 Mm) were added to a 96-well plate. The mixture was then shaken and reacted at room temperature for 30 min away from light. The absorbance was measured at 517 nm using a microplate reader. Ethanol was used instead of DPPH for the blank, and water was used instead of the sample for the control. The ability to scavenge DPPH radicals was calculated according to the following equation:

The scavenging activity of DPPH (%) = [1 − (A_sample_ − A_blank_)/A_control_] × 100%

A_blank_, A_control_, and A_sample_ represent absorbance for blank, control, and sample, respectively. Trolox standard curve was applied to determine the Trolox equivalent antioxidant capacity. The results were expressed as Trolox equivalents (µmol TE/g sample).

#### 2.9.2. Fe^2+^-Chelating Activity Assay

The Fe^2+^-chelating activity was determined using the method of Zhao et al. [15] with some modifications. One mL of sample solution was blended with 0.1 mL of a FeCl_2_ solution (2 mM) and 4.5 mL of absolute ethanol while shaking. Then, 0.2 mL of a ferrozine solution (5 mM) was added to the solution previously mixed and left to react at room temperature for 10 min. An equal volume of distilled water was substituted for the sample to serve as a control group. The absorbance was measured by spectrophotometry at 562 nm and the Fe^2+^-chelating activity was calculated according to the following equation:

The Fe^2+^-chelating activity (%) = (1 − A_S_/A_C_) × 100%

A_S_ and A_C_ representing the sample and the blank absorbance, respectively.

#### 2.9.3. Reducing Power Assay

The reducing power of the ESM hydrolysates was determined according to Shi et al. [17]. After incubation, the absorbance of the mixed solution was determined at 700 nm. The increased absorbance of the reaction mixture indicated an increase in the energy added to the reduced iron.

### 2.10. Membrane Ultrafiltration

According to the method of Kang et al. [30], HEMH solutions were added to ultrafiltration centrifuge tubes with 30 kDa, 10 kDa, and 3 kDa cut-off filters, and then centrifuged at 3000× *g* for 15 min. Peptide fractions labeled HEMH-I (MW < 3 kDa), HEMH-II (MW 3–10 kDa), HEMH-III (MW 10–30 kDa), and HEMH-IV (MW > 30 kDa) were obtained and freeze-dried for later use.

### 2.11. Effects of HEMH-I on H_2_O_2_-Induced SW1353 Cells

#### 2.11.1. Cell Culture

SW1353 human chondrocytes were purchased from the Chinese Academy of Sciences. The cells were cultured in DMEM (Procell, Wuhan, China) supplemented with 10% FBS and 1% penicillin/streptomycin and glutamine and incubated in a humidified incubator at 37 °C with 5% CO_2_. The cells were passaged every three days.

#### 2.11.2. Cytotoxicity Analysis of HEMH-I and H_2_O_2_ on SW1353 Cells

Cell viability was measured using the cell counting kit-8 (CCK-8) assay (Biosharp, Hefei, China). The SW1353 cells (2 × 10^4^ cells/mL) were incubated for 24 h and then exposed to HEMH-I dissolved in DMEM at a concentration of 0.25, 0.5, 0.75 mg/mL for 24 h. According to the method provided by the manufacturer, 100 μL of CCK-8 solution was added to each well. After incubation at 37 °C for 1 h, the absorbance was determined at 450 nm.

H_2_O_2_ was used to treat SW1353 cells to establish an in vitro OA model. SW1353 cells (2 × 10^4^ cells/mL) were seeded into 96-well plates and incubated for 24 h. The cells were cultured in DMEM for 24 h and then exposed to H_2_O_2_ (100, 200, 400, 600, 800 μM) for 60 min. After rinsing the cells once with PBS, cell viability was determined by the CCK-8 assay.

#### 2.11.3. Cytoprotective Effect of HEMH-I on H_2_O_2_-Damaged SW1353 Cells

SW1353 cells were seeded into 96-well plates (2 × 10^4^ cells/well) and cultured with 0.125, 0.25, 0.5, 0.75 mg/mL HEMH-I for 24 h. Next, the cells were exposed to 200 μM H_2_O_2_ for 60 min and washed with PBS solutions. The cytoprotection effect of HEMH-I on H_2_O_2_-damaged SW1353 cells was measured by a CCK-8 assay.

#### 2.11.4. Determination of Intracellular ROS

The level of ROS in SW1353 cells was determined using a DCFH-DA probe to evaluate the oxidative stress in cells. According to the method of Wang et al. [31], cells were pretreated with HEMH-I (0.25, 0.5 and 0.75 mg/mL) for 24 h, then exposed to H_2_O_2_ (200 μM) for 60 min. After two washes with PBS, SW1353 cells were incubated in the dark for 30 min with 100 μL 2’,7’-dichlorofluorescein diacetate (DCFH-DA) (Nanjing Jian cheng Bioengineering Institute, Nanjing, China) at a concentration of 10 μM. A fluorescence microscope (Olympus X71, Tokyo, Japan) was used to observe the fluorescence images, and the fluorescence intensity was quantified using the Image-J software.

#### 2.11.5. Western Blotting Analysis

SW1353 cells (4.5 × 10^5^ cells/well) were plated in 6-well plates and incubated for 24 h, followed by treatment with different concentrations of HEMH-I (0.25 and 0.75 mg/mL) for 24 h and then treatment with H_2_O_2_ (200 μM) for 60 min. Next, the cells were lysed with RIPA lysis buffer containing PMSF and phosphatase inhibitors. The lysate was collected and centrifuged. The supernatant was then collected to determine the protein concentration. A total of 40 μg of protein was separated by 12% SDS-PAGE and electrotransferred to PVDF membranes, and the membranes were sealed with 5% skim milk for 2 h. The membranes were then incubated with primary antibodies (rabbit polyclonal antibodies) against Keap1 (1:3000; Proteintech, 10503-2-AP, Wuhan, China), Nrf2 (1:1000; Affinity Bioscience, AF0639, Cincinnati, OH, USA), HO-1 (1:2000; Proteintech, 10701-1-AP, Wuhan, China), SOD1 (1:2000; Invitrogen, PA5-27240, Waltham, MA, USA), IL-6 (1:1000; Affinity Bioscience, DF6087, Cincinnati, OH, USA), MMP-3 (1:1000; Affinity Bioscience, DF6334, Cincinnati, OH, USA), MMP-13 (1:1000; Affinity Bioscience, AF5355, Cincinnati, OH, USA), and type II collagen (1:1000; Bioss, bs-0709R, Woburn, MA, USA) for 12 h at 4 °C, followed by HRP conjugated goat anti-rabbit secondary antibodies (1:10,000; Boster, BA1054, Wuhan, China) for 2 h at 25 °C. The immunoblots were performed with ECL detection reagent (Applygen, Beijing, China), and the bands’ intensity was determined using the ImageJ software’s optical density method.

### 2.12. Statistical Analysis

All treatments in the experiment were performed at least in triplicate. Significant differences (*p* < 0.05) in results were determined by analysis of variance (ANOVA) followed by Duncan’s multiple range test or by Student’s *t*-test for two-group comparisons applying Statistix software 9.0 (Analytical Software, Tallahassee, FL, USA). All results of the experiment are reported as mean ± standard deviation (SD).

## 3. Results and Discussion

### 3.1. Structure and Chemical Composition of ESM from Two Sources of Chicken Eggs

It is well recognized that the structure and composition of protein substrates affect the efficiency of enzyme hydrolysis and the activity of hydrolysis products [32,33]. Therefore, it was necessary to characterize the hatched ESM as a novel source of protein material prior to the hydrolysis process. The fresh ESM, a well-studied material to generate bioactive peptides, was used as a control.

#### 3.1.1. Electron Microscopic Scanning of ESM

**Figure 1:**
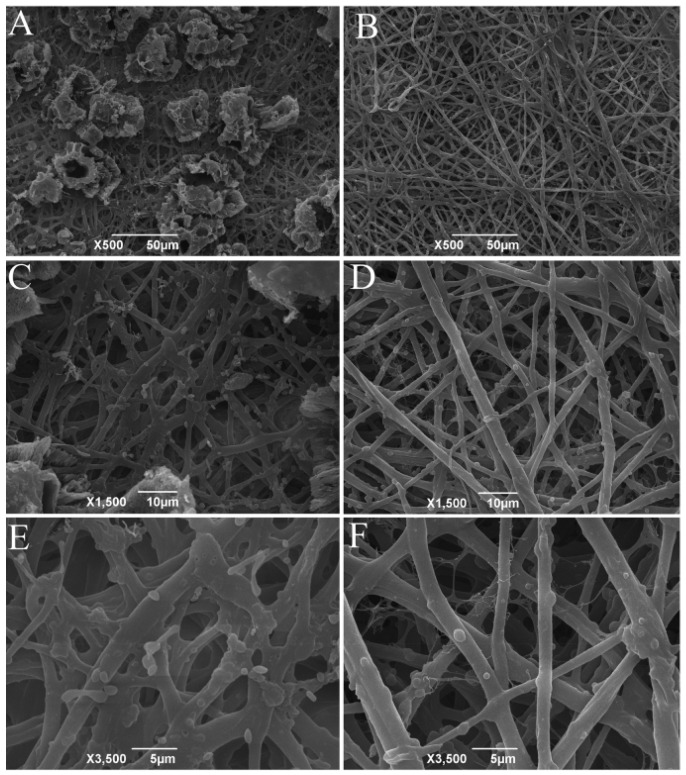
Scanning electron micrographs of hatched ESM (**A**,**C**,**E**) and fresh ESM (**B**,**D**,**F**) morphologies. Scale bars: (**A**,**B**) = 50 µm; (**C**,**D**) = 10 µm; (**E**,**F**) = 5 µm. ESM, eggshell membrane.

The scanning electron micrographs (SEM) of the hatched and fresh ESM are shown in Figure 1. The SEM pictures revealed that the main structure for both ESM is a meshwork of highly cross-linked fibers, in agreement with previous studies establishing that ESM is formed by interlaced insoluble protein fibers [34,35]; however, the surface morphology of the hatched ESM differs from that of the fresh ESM. Many aggregates, defined as the residual mammillary cones [36], were deposited on the surface of the outer hatched ESM fibers in a quasi-periodic pattern, consistent with previous studies [37]. In contrast, no cone tips remained on the fresh ESM fibers. This difference may be due to the calcium resorption that occurred during embryonic development, resulting in the complete detachment of the hatched ESM with attached mammillary cones from the eggshells, which led to the residual mammillary cones existed on the hatched ESM [37,38]; whereas for fresh eggs, the cones were removed together with the eggshell when the membranes were mechanically pulled from the shell interior [37]. Since the mammillary cones are rich in proteins [36], the hatched ESM should also contain some specific mammillary cone proteins, making its protein composition different from that of the fresh ESM.

In addition, the difference between the hatched and fresh ESM may also be related to the incubation process. Rath et al. [22] reported that embryonic development may induce some dynamic changes in the egg proteome and alter the membrane structure to facilitate the hatching process. An increase in oxygen permeability of ESM has been reported during incubation [39], which may lead to a decrease in density of the hatched ESM structures. In addition, the epithelial cells in the chorioallantoic membrane secrete protons to dissolve the calcium reserve [36,38]. The released protons may dissolve the acid-soluble proteins of the ESM, leading to a decrease in the density of the three-dimensional structure of the hatched ESM [40]. In line with these previous findings, the network of the hatched ESM appeared different and much flatter in the present study, probably due to the physical and physiological changes in the ESMs occurring during the embryonic development [39]. Less intensity of insoluble protein fibers may lead to a better efficiency of enzymatic hydrolysis, which will be validated in the next part.

#### 3.1.2. Proximate Composition of ESM

The chemical composition of the hatched and fresh ESM was established, where the results for protein, ash, calcium, and saccharide were expressed as g/100 g dry matter (Table 1). Protein was the main component for both ESMs, ranging from 92.98 to 96.00, in agreement with the 88–96% protein range of fresh ESM in dry matter reported by Huang et al. [16]. Due to their high protein content, ESM can be directly used as a protein material to produce bioactive peptides without any additional protein extraction step [15].

Some differences in composition were noted between the two ESMs. The hatched ESM contained significantly lower protein levels than the fresh ESM. This may be due to multiple factors, including the acidic environment that dissolves eggshell calcium during incubation [21], potentially leading to the dissolution of acid-soluble proteins in the ESM and their absorption by the embryo. It was reported that clusterin, a major component of the egg proteome in fresh ESM, was potentially absorbed completely during incubation [22]. The higher ash and calcium contents in the hatched ESM were consistent with the observations of SEM.

#### 3.1.3. Amino Acid Composition of ESM

The amino acid compositions of the fresh and hatched ESMs are shown in Table 2. Glu, Asp, Cys, and Pro were the main constituents of the two ESMs. A previous study of fresh ESM reported a similar amino acid composition [41]. Despite some similarities, there were significant differences in amino acid composition between the two ESMs, especially in Cys, Pro, Ala, Leu, and Lys content. In particular, Cys and Pro have been reported as the key constituent amino acids of cysteine-rich eggshell membrane proteins (CREMPs) and collagen, the structural proteins in ESM [23,41]. The content in Cys and Pro were noticeably higher in fresh ESM than in hatched ESM, implying a much stronger protein network structure in the fresh ESM, which is consistent with our observation of the SEM. The content in Leu, Ala, and Lys, known as hydrophobic amino acids (HAA) [42], were higher in the hatched ESM than the fresh ones. HAA play an important role in scavenging free radicals by increasing the solubility of peptides in the lipid phase and facilitating the interaction between peptides and free radicals [8]. The difference in amino acid profiles between the two ESMs indicated that their hydrolysates will differ both in nutritional values and bioactivities. The hatched and fresh ESMs contain all essential amino acids (EAA), accounting for 30.57% and 29.04%, respectively, values similar to or even higher than those for many other food source proteins, including walnut protein (26.98%), and cashew protein (29.92%) [28,43].

The above results further suggested that the hatched ESM can potentially serve as a novel protein source, with a balanced amino acid composition and high nutrition value, to prepare hydrolysates for food products. The different structure and chemical composition between the two sources of ESMs were expected considering the qualitative and quantitative changes in ESM during incubation and passive storage [21,22,44]. These differences might also give rise to significantly different proteolytic properties and hydrolysate bioactivities. Therefore, we further analyzed the hydrolytic properties of the hatched ESM as a novel raw material to obtain bioactive peptides and studied the activity of their hydrolysates, using the fresh ESM as a control.

### 3.2. Enzymatic Hydrolysis of ESM 

Enzymatic hydrolysis is considered the most efficient method to produce bioactive peptides from protein precursors, allowing to retain their full nutritional value [45], and avoiding the presence of residual organic solvents or toxic chemicals in the final product, thus making it a widely used practice in the food industry [46]. In this experiment, alcalase, a widely used protease to prepare bioactive peptides from natural protein sources [2,29], was chosen to prepare ESM protein hydrolysates since our previous studies [15,16] demonstrated its value to generate fresh ESM hydrolysates with strong antioxidant properties, which was better than those of hydrolysates obtained with other commonly used proteases [15].

#### 3.2.1. Degree of Hydrolysis (DH)

DH is an essential parameter of hydrolysis, for it can affect the size of peptides, and thus regulating their biological activities [46,47]. In addition, a high DH indicates a high peptide content obtained during hydrolysis, which increased the potential for recovering the protein source for use as a food additive [46].

**Figure 2:**
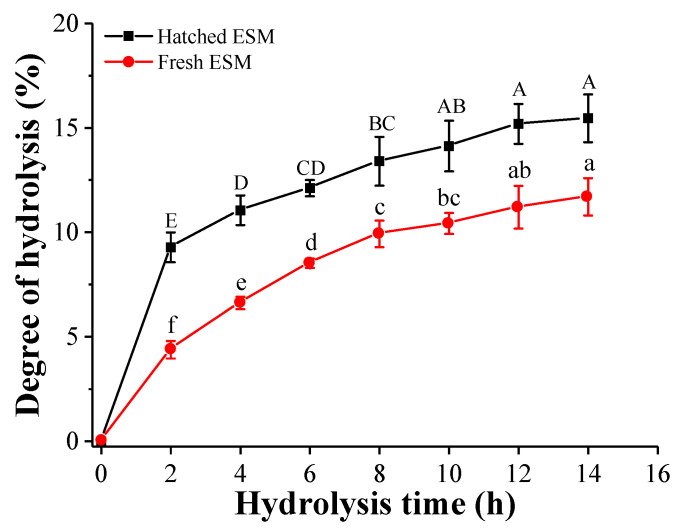
The degree of hydrolysis of the hatched ESM (■) and fresh ESM (●) hydrolyzed under the same enzymatic hydrolysis condition. Values are expressed as mean ± SD (*n* = 3). Different letters in the hydrolysis kinetic curve of hatched ESM (A–E) and fresh ESM (a–f) indicate significant differences (*p* < 0.05).

As shown in Figure 2, the hydrolysis kinetics of the hatched and fresh ESM obtained with alcalase reveal an increase in DH with time, corresponding to the gradual release of peptide fragments during hydrolysis. The DH curve for both ESMs is a typical hyperbolic trend with a high rate during the initial stage of hydrolysis followed by a stationary phase until reaching a steady state after 12 h. Similar DH curves were also observed for the hydrolysates of different protein sources such as Bambara bean and rice bran [47,48]. The hatched ESM treated under the same enzymatic hydrolysis conditions possessed a markedly higher degree of hydrolysis (9.28 to 15.46%) during the entire hydrolysis period when compared with the fresh ESM (4.38 to 11.70%), indicating a higher hydrolysis efficiency of the hatched than the fresh ESM.

The nature of the protein substrates is known to affect the release of bioactive peptides [47]. Under the same hydrolysis conditions, the difference in DH between the two ESMs may therefore be attributed to their different chemical composition and structure. These DH results were consistent with the structural differences in SEM and amino acid analysis, indicating that the hatched ESM potentially possessed a weaker protein network structure, thus more conducive to enzymatic hydrolysis. The protein hydrolysates can be categorized based on DH values. When the DH value is greater than 10%, it represents high hydrolysis, and highly hydrolyzed protein hydrolysates offer more potential to be used as nutritional supplements or nutraceuticals [4]. Therefore, the hatched ESM hydrolysates obtained in this study were considered highly hydrolyzed and possessed potential applications in functional foods.

#### 3.2.2. Molecular Weight Distributions of ESM Hydrolysates

**Figure 3:**
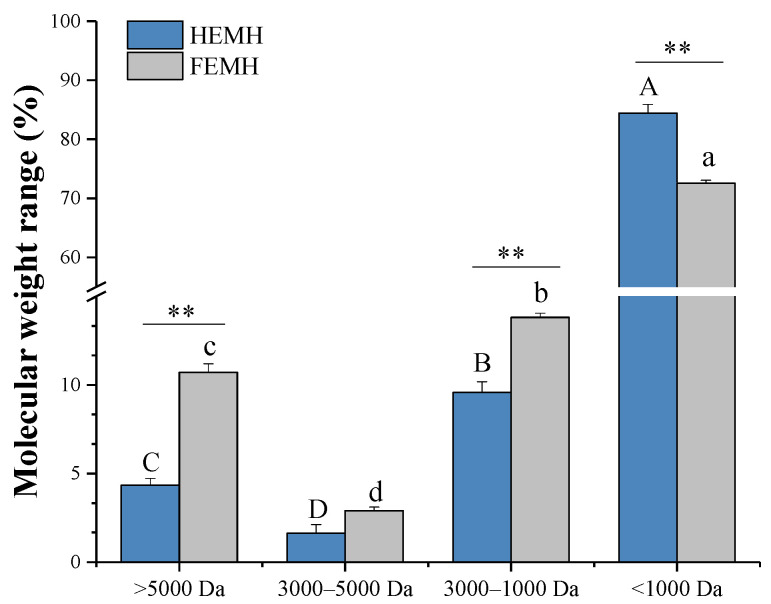
Molecular weight (MW) distribution of hydrolysates of hatched eggshell membrane (HEMH) and fresh eggshell membrane (FEMH). Values are expressed as mean ± SD (*n* = 3). Different letters in FEMH (A–D) or HEMH (a–d) with MW > 5000 Da, 3000–5000 Da, 1000–3000 Da, and <1000 Da mean significant differences for different MW comparisons (*p* < 0.05), and the significance levels of ** *p* < 0.01 are for FEMH versus HEMH at the same MW.

The molecular weight (MW) distributions of hydrolysates from the two ESMs were determined by HPLC. As shown in Figure 3 and Figure S2, both ESM hydrolysates were mainly composed of low MW peptides (<3000 Da), confirming that small molecular bioactive peptides were released from both ESMs during the enzymatic process. However, the MW distribution of the FEMH and HEMH also exhibited significant differences. The HEMH contained more peptides with small MW (<1000 Da), up to 84.44%, than those of the FEMH, up to 72.58% (*p* < 0.01). The higher content of low-MW peptides in the HEMH is consistent with the higher DH found in the HEMH.

Similarly, Habinshuti et al. [49] and Zhang et al. [50] reported that higher DH values produce low MW peptides. It is well known that hydrolysates containing a higher percentage of lower MW peptides tend to have a higher antioxidant activity [51]. In addition, Mune et al. [47] reported that peptides with small MW (<1000 Da) presented the highest biological activity. Thus, the higher content of low MW peptides in the HEMH may result in higher biological activity than in the FEMH.

#### 3.2.3. Amino Acid Composition of HEMH and FEMH Fractions

The amino acids present in the HEMH and FEMH are presented in Figure S3. Since the biological activity of protein hydrolysates mainly depends on the type and amount of amino acids in their protein sequences [28,52], the amino acid profiles were analyzed. Generally, the HEMH had an amino acid profile similar to the FEMH but differed in the content of various amino acids. Both profiles showed a high level of hydrophobic amino acids (Leu, Val, Ala, Pro, Phe, His, Trp, Gly, Lys, and Ile) and aromatic amino acids (Phe, Trp, and Tyr), previously reported to contribute to the antioxidant and other bioactive properties of peptides [8]. Asp and Gly were the most abundant amino acids of HEMH and FEMH, with contents of 6.93–7.07 g/100 g protein and 5.57–5.75 g/100 g protein, respectively. Jain and Anal [14] and Shi et al. [52] have reported similar results for the hydrolysates derived from fresh ESM. These two amino acids are recognized as strong electron donors or chelators for metal ions, which can improve the antioxidant potential of hydrolysates [53]. In addition, compared to the FEMH, the HEMH presented significantly (*p* < 0.05) higher contents of Met, Lys, and Try, which have been reported to have antioxidant effects [54]. Finally, a higher content of EAA (17.02 g/100 g) was observed in the HEMH than in the FEMH (15.95 g/100 g). 

Overall, the above characterization results of hydrolysates from hatched ESM, using the hydrolysates of the well-studied fresh ESM as a control, indicated that the hydrolysates from hatched ESM have a high potential for antioxidant and nutritional properties. In the following experiments, the peptide composition and antioxidant activity of HEMH were further analyzed.

#### 3.2.4. Nano-LC-ESI-MS/MS Analysis of HEMH

**Figure 4:**
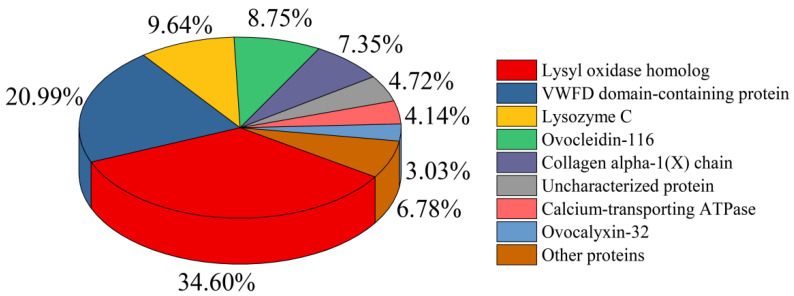
Percent distribution of identified peptides according to their parent proteins measured by Nano-LC-ESI-MS/MS.

It is well known that the biological activity of a specific protein hydrolysate is closely related to its peptide composition [2]. In order to quantitatively identify the HEMH peptides, the nano-LC-ESI-MS/MS analysis using Maxquant software was conducted. As shown in the total ion chromatogram (Figure S4), the LC-MS/MS analysis was completed within 60 min, showing typical peaks corresponding to specific peptides, and 249 peptide sequences derived from HEMH were identified. Figure 4 presents the percentage distribution of peptides from specific parent proteins, including lysyl oxidase homolog (34.60%), VWFD domain-containing protein (21.00%), lysozyme C (16.24%), ovocleidin-116 (8.75%), collagen alpha-1(X) chain (7.35%), and calcium-transporting ATPase (4.14%). This result indicated that most abundant peptides present in HEMH are derived from the parent protein, Lysyl oxidase homolog. It is an enzyme that catalyzes the covalent linkage between collagen and elastin, and plays a critical role in the formation of ESM fiber as a major protein component of ESM [23,41]. Other parent proteins are also reported to have specific biological activity, such as lysozyme C, which is known to possess antibacterial and anti-inflammatory activities [12]. In addition, to understand the potential bioactivity of the identified peptides, 10 major peptide sequences (854–1385 Da) in HEMH were evaluated for bioactivity using PeptideRanker (Table S1), a server for the prediction of bioactive peptides based on a novel N-to-1 neural network and scores greater than 0.5 were considered bioactive [15,24]. The results showed that 60% of the 10 major peptides had scores above 0.5, indicating potentially high biological activity of HEMH.

### 3.3. The Antioxidation Activity of HEMH and Ultrafiltration Fractions

In chemical antioxidant activity assays, the antioxidation of peptides or protein hydrolysates depends on their free radical scavenging ability, metal ion chelating ability, and reducing ability [52,55]. The DPPH free radical scavenging assay is an antioxidant activity evaluation method based on the electron transfer mechanism and is commonly used to assess the antioxidant capacity of natural compounds [42]. Chelation of metal ions is an essential pathway for antioxidant action, as Fe^2+^ ions are catalysts for the chain reaction of lipid peroxidation, leading to the oxidation of unsaturated lipids [56]. The reducing power represents the ability to contribute electrons or hydrogen, and the ferric ion reducing power determination involves a redox reaction in which the compound reduces Fe^3+^ to Fe^2+^ [50].

To further investigate their antioxidant activities, our study determined the DPPH radical scavenging activity, Fe^2+^-chelating activity, and Fe^3+^-reduction capacity of the HEMH and FEMH. As shown in Table 3 the HEMH possessed better antioxidant activities in DPPH radical scavenging ability (153.51 ± 12.63 μmol TE/g), Fe^2+^-chelating activities (80.11 ± 0.30%), and reducing power (0.67 ± 0.005) than the FEMH (51.93 ± 2.47 μmol TE/g, 56.51 ± 1.17%, and 0.10 ± 0.01%, respectively). These results were consistent with the finding that the HEMH exhibited a higher DH value and a higher content in small molecule peptides, when compared to the FEMH. 

In order to investigate the relationship between the antioxidant activity and the MW of the HEMH, as well as to obtain a purified fraction with a higher bioactivity, the antioxidant activity of ultrafiltration fractions of the HEMH with different MW was further determined. It was found that the peptide fraction with the smallest MW (HEMH-I, MW < 3 kDa) had the highest antioxidant activity, whereas the HEMH-IV (MW > 30 kDa), the largest MW fraction, exhibited the weakest antioxidant effect, in agreement with our previously reported results [24]. Several studies have reported that the antioxidant activity of peptides is related to MW distribution [42,57], and that lower MW peptides are considered to have higher antioxidant activity than higher MW peptides [58]. Altogether, the HEMH has a higher DH value, a higher amount of low molecular weight peptides, and a higher antioxidant activity than the FEMH under the same enzymatic hydrolysis conditions, indicating that the hatched ESM showed more potential than the fresh ESM to release antioxidant peptides. The HEMH-I fraction with the highest antioxidant activity was then evaluated for its protective effects against oxidative stress in SW1353 chondrocytes.

### 3.4. Cytoprotective Effect of HEMH-I on H_2_O_2_-Induced SW1353 Human Chondrocytes

SW1353 human chondrocytes are one of the most common and established cell lines used for substitution of primary chondrocytes [59]. H_2_O_2_ is an important factor involved in the pathogenesis of OA. In chondrocytes, H_2_O_2_ can induce the cellular production of ROS, inhibit proteoglycan synthesis and promote ECM degradation, causing the expression of inflammatory cytokines and matrix metalloproteinases (MMPs) [60]. Therefore, H_2_O_2_-induced SW1353 human chondrocytes are an representative in vitro OA model [3], replacing articular chondrocytes and animal models in early stages of development of novel OA therapeutics while meeting the need for a simplified approach to understand the pathophysiology of OA [59,61]. In this study, we used H_2_O_2_-induced SW1353 cells to investigate the potential role of HEMH-I in protecting chondrocytes against cellular oxidative stress.

#### 3.4.1. Effects of HEMH-I on Cell Viability of H_2_O_2_-Induced SW1353 Human Chondrocytes

**Figure 5:**
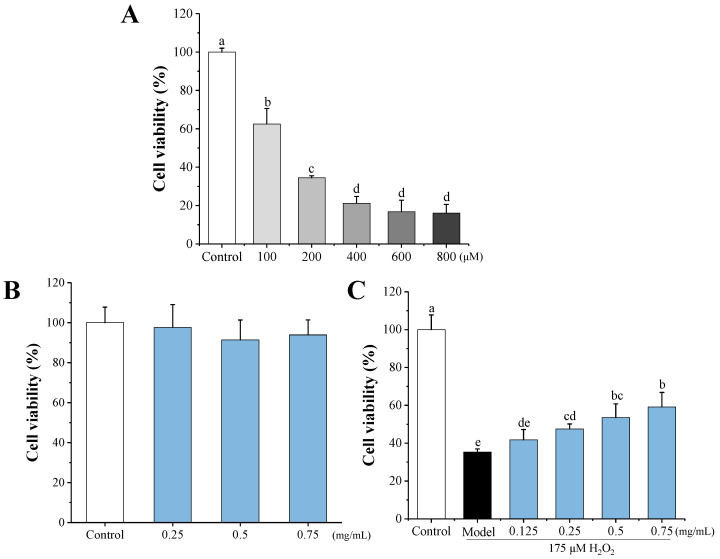
Effects of HEMH-I on the viability of SW1353 cells; (**A**) Effects of H_2_O_2_ at different concentrations on SW1353 cell viability; (**B**) Cytotoxicity of HEMH-I; (**C**) Protective effect of HEMH-I on the viability of SW1353 cells under oxidative stress conditions. Note: values are expressed as mean ± SD (*n* = 5). Means annotated with different letters for the same parameter are significantly different (*p* < 0.05).

In order to obtain an insight into the response of SW1353 chondrocytes to H_2_O_2_ treatment, as well as to establish the optimal conditions for H_2_O_2_ induction, cells were cultured in the presence of different concentrations of H_2_O_2_ (100, 200, 400, 600, and 800 μM) for 1 h. As seen in Figure 5A, the cell viability decreased to 16.01–62.57% after exposure to H_2_O_2_, indicating that H_2_O_2_ has a dose-dependent toxic effect on SW1353 cells. The 200 μM H_2_O_2_ concentration was selected to induce oxidative stress. In addition, the cytotoxic effect of HEMH-I (0.25, 0.5 and 0.75 μg/mL) on SW1353 cells was determined by CCK-8 assay. Figure 5B demonstrated no toxicity on SW1353 cells for HEMH-I at concentrations up to 0.75 μg/mL. To evaluate the protective effect of HEMH-I against oxidative stress in chondrocytes, the effects of HEMH-I at the level of 0.125, 0.25, 0.5, and 0.75 mg/mL on the cell viability of H_2_O_2-_damaged SW1353 cells were measured. As displayed in Figure 5C, all concentrations of HEMH-I could significantly reverse the H_2_O_2_-induced injury in SW1353 cells (*p* < 0.05), and the viability of the cells was increased by 34.59–51.32% after HEMH-I treatment. This result indicated that HEMH-I possessed a protective effect against H_2_O_2_-induced cell damage in the in vitro OA model.

#### 3.4.2. Effects of HEMH-I on ROS Levels in H_2_O_2_-Induced SW1353 Human Chondrocytes

**Figure 6:**
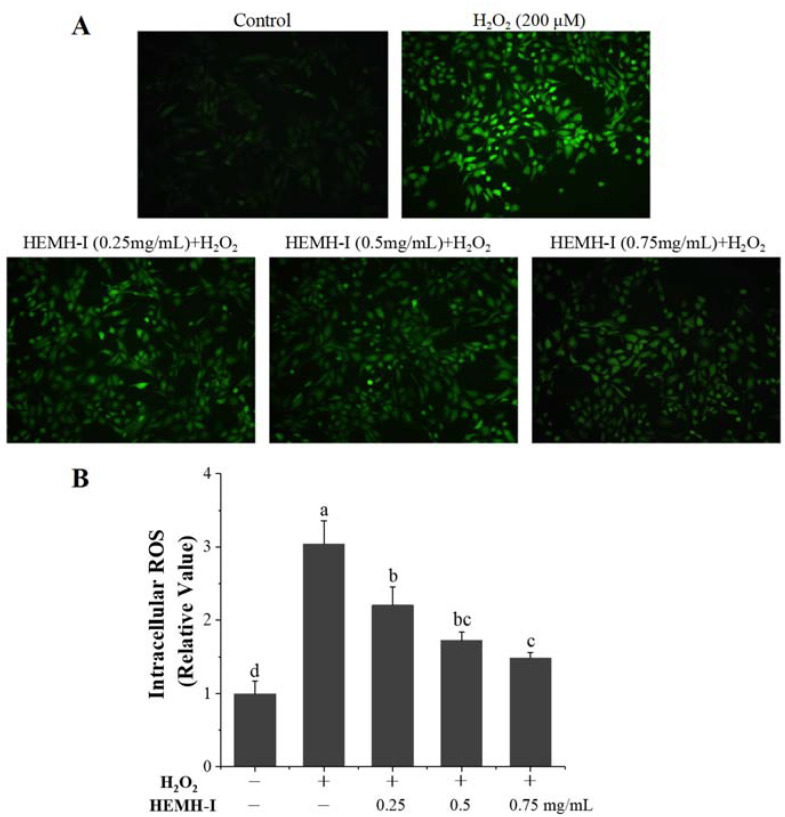
Effect of HEMH-I on ROS generation in the H_2_O_2_-induced SW1353 cells. (**A**) Fluorescence micrograph; (**B**) Fluorescence intensity of different groups. Values are expressed as mean ± SD (*n* = 5). Different letters (a–d) mean significant differences (*p* < 0.05).

The oxidative stress levels in cells can be assessed using ROS measurement. Inhibiting cellular damage to chondrocytes by regulating ROS production is an important therapeutic strategy [3]. In this study, the ROS levels of different treatment groups were determined by applying DCFH-DA fluorescence dye. As shown in Figure 6A, the H_2_O_2_ model group exhibited the brightest fluorescent signal, while the control group exhibited the lowest fluorescent signal, indicating that H_2_O_2_-induced oxidative stress in SW1353 cells by accumulating ROS. Compared with the H_2_O_2_ damage group, the fluorescence level of SW1353 cells was significantly reduced after pretreatment with different concentrations of HEMH-I. The fluorescence intensities of all groups were also quantified using Image J software (Figure 6B). The ROS level of the model group was 2.04-fold higher than that of the control group (*p* < 0.05). Meanwhile, the pretreatment with HEMH-I (0.25, 0.5 and 0.75 mg/mL) significantly reduced the intracellular fluorescence intensity of SW1353 by 28%, 43%, and 51%, respectively (*p* < 0.05), suggesting that HEMH-I could dose-dependently reduce H_2_O_2_-induced ROS production in SW1353 cells and thus exerting protection against cellular oxidative stress damage. 

#### 3.4.3. Effects of HEMH-I on Antioxidant Enzymes and Inflammatory Factor Expression in SW1353 Human Chondrocytes

As the first line of defense against ROS, superoxide dismutase (SOD) plays an important role in maintaining the dynamic balance of oxidation and antioxidation in the body [62]. IL-6 is one of the most important inflammatory markers in OA and represents the level of oxidative stress-induced inflammation in chondrocytes [63]. In order to evaluate the effect of HEMH-I on oxidative stress and inflammatory response in an in vitro OA model, the expression levels of SOD1 and IL-6 in SW1353 cells were determined using western blot analysis.

**Figure 7:**
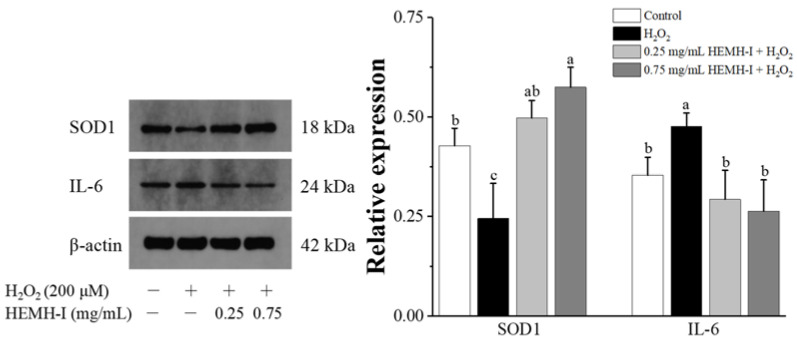
Effect of HEMH-I on expressing antioxidant enzyme SOD1 and inflammatory factor IL-6 in SW1353 chondrocytes. Values are expressed as mean ± SD (*n* = 3). Means annotated with different letters (a–c) for the same parameter are significantly different (*p* < 0.05).

The results are shown in Figure 7. H_2_O_2_ treatment significantly reduced the expression of SOD1 and promoted the expression of the inflammatory factor IL-6 (*p* < 0.05), indicating that H_2_O_2_-induced an increase in oxidative stress and inflammation in SW1353 cells. In contrast, 0.25 mg/mL and 0.75 mg/mL of HEMH-I treatment markedly increased the expression of SOD1 by 1.03- and 1.37-fold, respectively, when compared with the model group (*p* < 0.05). Meanwhile, low-dose and high-dose HEMH-I significantly reduced the H_2_O_2_-induced IL-6 expression by 48.38% and 55.85%, respectively (*p* < 0.05). The above results indicated that HEMH-I could effectively reduce cellular oxidative stress and inflammatory responses in the H_2_O_2_-induced SW1353 human chondrocytes.

#### 3.4.4. Effect of HEMH-I on the Expression of Collagen II, MMP3, and MMP13 in SW1353 Human Chondrocytes

Increased ROS in chondrocytes can lead to excessive degradation of the ECM, which plays a central role in the progression and development of OA [31]. Type II collagen is a major constituent of articular cartilage [5]. The overexpression of protein hydrolases, including MMPs, by chondrocytes leads to excessive degradation of type II collagen and other ECM proteins, which is a pivotal event in the pathogenesis of OA [64]. As one of the important MMPs, MMP3 can activate other MMPs (MMP2, MMP9, MMP13, etc.) from the proMMPs and thus plays a key role in the progression of ECM degradation [3,65]. In addition, MMP13 is a major enzyme hydrolyzing type II collagen [60]. Accordingly, the effects of HEMH-I on type II collagen, MMP3 and MMP13 expression in H_2_O_2_-induced SW1353 cells were evaluated to investigate the protective role of HEMH-I against ECM degradation.

**Figure 8:**
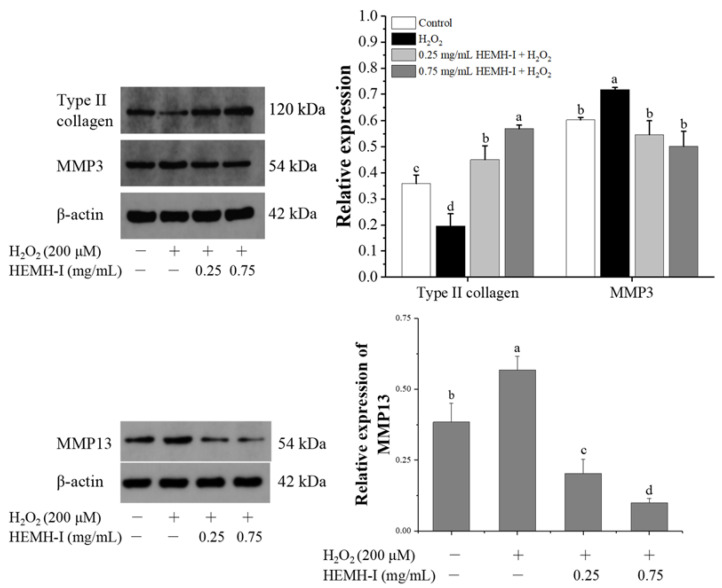
Effect of HEMH-I on the expression of Type II collagen, MMP3 and MMP13 in SW1353 chondrocytes. Values are expressed as mean ± SD (*n* = 3). Means annotated with different letters (a–d) for the same parameter are significantly different (*p* < 0.05).

As shown in Figure 8, the exposure to H_2_O_2_ significantly increased the expression of MMP3 and MMP13 while decreasing the level of type II collagen, indicating that oxidative stress induced by H_2_O_2_ stimulated ECM degradation in SW1353 human chondrocytes, in agreement with Kim et al. [3] who reported that H_2_O_2_-induced ROS could reduce the role of chondrocytes in the formation and maintenance of cartilage ECM. However, HEMH-I treatment at 0.25 and 0.75 mg/mL significantly decreased MMP3 and MMP13 expression compared to the model group. Analogous results for MMP3 and MMP13 expression were observed in previous studies of bioactive substances ameliorating ECM degradation in chondrocytes [66,67]. Additionally, type II collagen protein levels were increased by 1.29-fold and 1.90-fold, respectively. These results suggested that HEMH-I could attenuate ECM degradation by effectively regulating type II collagen, MMP3 and MMP13 expression in SW1353 chondrocytes under oxidative stress.

#### 3.4.5. Effect of HEMH-I on the Activation of Keap1/Nrf2/HO-1 Pathway of SW1353 Human Chondrocytes

The Keap1/Nrf2/HO-1 signaling pathway is an essential regulatory pathway for cellular resistance to oxidative stress induced by various exogenous and endogenous factors [25,68]. In this pathway, when cells are under stressed conditions or in the presence of certain bioactive molecules, nuclear factor erythroid 2-associated factor 2 (Nrf2) will separate from its inhibitor Kelch-like ECH-associated protein 1 (Keap1) and bind to the antioxidant response element, initiating the expression of heme oxygenase-1 (HO-1) and leading to the activation of the defense system against oxidative stress [65]. Recently, this pathway has been identified as a novel therapeutic target against OA [1,6]. Many studies have supported the important role of Nrf2 and HO-1 expression in this signaling pathway to reduce oxidative stress, inflammatory response, and cartilage degradation in OA [69,70]. Our previous study has shown that HEMH-I can regulate the Keap1/Nrf2 signaling pathway to reduce oxidative stress in RAW264.7 cells [24]. However, whether HEMH-I exerts a regulatory effect on Keap1/Nrf2/HO-1 pathway in OA chondrocytes in vitro is not known. Therefore, the expression of Keap1, Nrf2, and HO-1, the key proteins of this signaling pathway, were further determined in SW1353 human chondrocytes.

**Figure 9:**
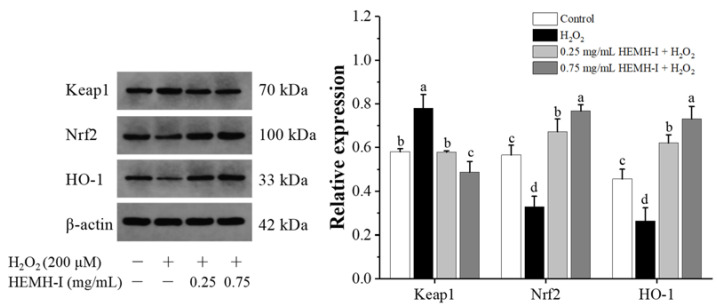
Effect of HEMH-I on the expression of Keaps1, Nrf2, and the downstream antioxidant enzyme HO-1 in SW1353 human chondrocytes. Values are expressed as mean ± SD (*n* = 3). Means annotated with different letters (a–d) for the same parameter are significantly different (*p* < 0.05).

As shown in Figure 9, H_2_O_2_ treatment resulted in a 34.24% increase in the expression of Keap1 and a significant decrease in the expression of Nrf2 and HO-1 in SW1353 chondrocytes when compared to the control group (*p* < 0.05). Both low-dose and high-dose HEMH-I treatments significantly decreased the expression of Keap1 while significantly increasing the expression of Nrf2 and HO-1, the downstream antioxidant enzyme of this pathway, by 1.35-fold and 1.77-fold, respectively (*p* < 0.05).

These results indicated that the mechanism exhibited by HEMH-I to protect chondrocytes against oxidative damage was related to the activation of the antioxidant signaling pathway Keap1/Nrf2/HO-1. Similar studies of many natural bioactive components such as allicin, sulforaphane, and lycopene were reported to reduce oxidative stress, decrease inflammatory marker expression, and increase chondrogenic markers in the H_2_O_2_-induced chondrocytes by activating this pathway [3,67].

Taken together, the cell experiment results indicated a novel beneficial role of HEMH-I in ameliorating oxidative stress, attenuating inflammatory factor expression, promoting type II collagen synthesis, and modulating the Keap1/Nrf2/HO-1 signaling pathways in H_2_O_2_-induced SW1353 chondrocytes. Notably, even though the SW1353 cells is widely used as an alternative to primary chondrocytes for in vitro OA study [3,59], our results could not be fully translated to primary OA chondrocytes due to the differences between SW1353 cells and human primary chondrocytes [66], which is a limitation of this study and warrants further investigation.

## 4. Conclusions

The hatched ESM is a high protein (92.98%) byproduct resource with significantly different structural properties and chemical composition from fresh ESM. It can be used to prepare antioxidant hydrolysates rich in low-MW peptides (94.63%, MW < 3 kDa) by enzymatic hydrolysis with alcalase. Compared with the fresh ESM, the hatched ESM potentially exhibited higher enzymatic hydrolysis efficiency and antioxidant activity of hydrolysates.

The hatched ESM hydrolysates with MW < 3 kDa, HEMH-I, showed the highest antioxidant activity and exerted cytoprotective activity to ameliorate the H_2_O_2_-induced oxidative stress, inflammatory response, and cartilage ECM degradation in SW1353 human chondrocytes, which may related to the activation of the cellular Keap1/Nrf2/HO-1 pathway.

This study provides a theoretical foundation for developing hatched ESM waste as a novel natural source to prepare antioxidant hydrolysates, as well as new insights into protective effect of HEMH-I against oxidative stress in chondrocytes. Further studies using primary chondrocytes and in vivo models are necessary to confirm the beneficial effects of HEMH-I on OA cartilage protection and the unique mechanisms behind them.

## Figures and Tables

**Table 1:** Proximate composition of the eggshell membrane (ESM) of hatched and fresh eggs (g/100 g).

Raw Material	Protein Content	Ash Content	Ca Content	Saccharide
Hatched ESM	92.98 ± 1.22 ^b^	3.62 ± 0.12 ^a^	0.66 ± 0.01 ^a^	2.1 ± 0.24 ^a^
Fresh ESM	96.00 ± 1.38 ^a^	2.96 ± 0.14 ^b^	0.45 ± 0.01 ^b^	1.81 ± 0.12 ^b^

Values are expressed as mean ± SD (*n* = 3). Means with different letters (a, b) for the same parameter are significantly different (*p* < 0.05).

**Table 2:** The composition of amino acids (g/100 g protein) in hatched and fresh ESM.

Amino Acid	Hatched ESM	Fresh ESM
Asp	7.69 ± 0.19	7.68 ± 0.13
Thr	4.74 ± 0.09 ^b^	4.97 ± 0.02 ^a^
Ser	5.08 ± 0.08	5.22 ± 0.02
Glu	10.29 ± 0.23	10.21 ± 0.14
Gly	5.57 ± 0.13	5.35 ± 0.13
Ala	3.22 ± 0.06 ^a^	2.61 ± 0.12 ^b^
* Cys	7.34 ± 0.12 ^b^	9.56 ± 0.44 ^a^
Val	3.45 ± 0.06	3.65 ± 0.12
Met	2.55 ± 0.07 ^a^	2.34 ± 0.03 ^b^
Ile	2.77 ± 0.06 ^a^	2.62 ± 0.04 ^b^
Leu	4.39 ± 0.19 ^a^	3.61 ± 0.06 ^b^
Tyr	1.44 ± 0.03 ^a^	1.16 ± 0.02 ^b^
Phe	1.73 ± 0.03 ^a^	1.32 ± 0.04 ^b^
His	2.95 ± 0.05 ^b^	3.18 ± 0.04 ^a^
Lys	3.16 ± 0.07 ^a^	2.47 ± 0.03 ^b^
Arg	5.6 ± 0.12	5.67 ± 0.01
* Pro	6.27 ± 0.09 ^b^	6.64 ± 0.08 ^a^
TAA	74.57 ± 1.6	73.48 ± 0.99
EAA	22.8 ± 0.58	21.34 ± 0.81
EAA/TAA	30.57 ± 0.12	29.04 ± 0.71

EAA, essential amino acids (Ile, Leu, Lys, Met, Phe, Thr, and Val); TAA, total amino acids; Values are expressed as mean ± SD (*n* = 3). Different superscripts (a–b) within the same row indicate significant differences (*p* < 0.05). “*” represents “the key constituent amino acids of CREMPs and collagen, the main structural proteins in eggshell membranes”.

**Table 3:** The antioxidant activities of fresh ESM hydrolysates (FEMH), hatched ESM hydrolysates (HEMH), and the ultrafiltration fractions of HEMH.

	DPPH Radical Scavenging Activity (μmol TE/g)	Fe^2+^ Chelating Activity (%)	Fe^3+^ Reducing Power (A700)
FEMH	51.93 ± 2.47 ^d^	56.51 ± 1.17 ^e^	0.10 ± 0.01 ^f^
HEMH	153.51 ± 12.63 ^a^	80.11 ± 0.30 ^b^	0.67 ± 0.005 ^b^
HEMH-I	156.5 ± 8.97 ^a^	83.26 ± 1.95 ^a^	0.864 ± 0.003 ^a^
HEMH-II	111.94 ± 9.85 ^b^	80.88 ± 0.13 ^b^	0.655 ± 0.007 ^c^
HEMH-III	116.89 ± 8.6 ^b, c^	70.87 ± 0.36 ^c^	0.51 ± 0.002 ^d^
HEMH-IV	98.45 ± 10.91 ^c^	61.21 ± 0.24 ^d^	0.47 ± 0.000 ^e^

Values are expressed as mean ± SD (*n* = 3). Means annotated with different letters (a–f) for the same parameter are significantly different (*p* < 0.05). HEMH-I, hatched ESM hydrolysate with MW < 3 kDa; HEMH-II, hatched ESM hydrolysate fraction with MW between 3–10 kDa; HEMH-III, hatched ESM hydrolysate with MW between 10–30 kDa; HEMH-IV, hatched ESM hydrolysate fraction with MW > 30 kDa.

## Data Availability

Data is contained within the article.

## Supplementary Materials

The following are available online at https://www.mdpi.com/article/10.3390/antiox11122428/s1, Figure S1: The flow chart of the research strategy of the present work. Figure S2: Molecular weight distribution of hatched eggshell membrane hydrolysates (A) and fresh eggshell membrane hydrolysates (B). Figure S3: The composition of amino acids (g/100 g) in hydrolysates of hatched ESM (HEMH) and fresh ESM (FEMH). The significance levels of * *p* < 0.05 and ** *p* < 0.01 are for FEMH versus HEMH at the same amino acid. Figure S4: Total ion chromatogram by Nano-LC-ESI-MS/MS. Table S1: The major peptides identified in HEMH by Nano-LC-ESI-MS/MS.

## References

[1] Ansari M.Y., Ahmad N., Haqqi T.M. (2020). Oxidative stress and inflammation in osteoarthritis pathogenesis: Role of polyphenols. Biomed. Pharmacother..

[2] Lv R., Dong Y., Bao Z., Zhang S., Lin S., Sun N. (2022). Advances in the activity evaluation and cellular regulation pathways of food-derived antioxidant peptides. Trends Food Sci. Technol..

[3] Kim E., Lee H., Jeong G. (2020). Cudratricusxanthone O Inhibits HO-Induced Cell Damage by Activating Nrf2/HO-1 Pathway in Human Chondrocytes. Antioxidants.

[4] Zhuang C., Wang Y., Zhang Y., Xu N. (2018). Oxidative stress in osteoarthritis and antioxidant effect of polysaccharide from angelica sinensis. Int. J. Biol. Macromol..

[5] González-Rodríguez M.L., Fernández-Romero A.M., Rabasco A.M. (2017). Towards the antioxidant therapy in Osteoarthritis: Contribution of nanotechnology. J. Drug Deliv. Sci. Technol..

[6] Sun K., Luo J., Jing X., Guo J., Yao X., Hao X., Ye Y., Liang S., Lin J., Wang G. (2019). Astaxanthin protects against osteoarthritis via Nrf2: A guardian of cartilage homeostasis. Aging.

[7] Poulet B., Beier F. (2016). Targeting oxidative stress to reduce osteoarthritis. Arthritis Res. Ther..

[8] Wen C., Zhang J., Zhang H., Duan Y., Ma H. (2020). Plant protein-derived antioxidant peptides: Isolation, identification, mechanism of action and application in food systems: A review. Trends Food Sci. Technol..

[9] Yang J., Sun-waterhouse D., Xiao Y., He W., Su G. (2019). Osteoarthritis-alleviating effects in papain-induced model rats of chicken cartilage hydrolysate and its peptide fractions. Int. J. Food Sci. Technol..

[10] Kumar S., Sugihara F., Suzuki K., Inoue N., Venkateswarathirukumara S. (2015). A double-blind, placebo-controlled, randomised, clinical study on the effectiveness of collagen peptide on osteoarthritis. J. Sci. Food Agric..

[11] Waheed M., Yousaf M., Shehzad A., Inam-Ur-Raheem M., Khan M.K.I., Khan M.R., Ahmad N., Abdullah, Aadil R.M. (2020). Channelling eggshell waste to valuable and utilizable products: A comprehensive review. Trends Food Sci. Technol..

[12] Xiao N., Huang X., He W., Yao Y., Wu N., Xu M., Du H., Zhao Y., Tu Y. (2021). A review on recent advances of egg byproducts: Preparation, functional properties, biological activities and food applications. Food Res. Int..

[13] Kulshreshtha G., Diep T., Hudson H.A., Hincke M.T. (2022). High value applications and current commercial market for eggshell membranes and derived bioactives. Food Chem..

[14] Jain S., Anal A.K. (2017). Production and characterization of functional properties of protein hydrolysates from egg shell membranes by lactic acid bacteria fermentation. J. Food Sci. Technol-Mysore.

[15] Zhao Q.C., Zhao J.Y., Ahn D.U., Jin Y.G., Huang X. (2019). Separation and Identification of Highly Efficient Antioxidant Peptides from Eggshell Membrane. Antioxidants.

[16] Huang X., Zhou Y.H., Ma M.H., Cai Z.X., Li T. (2010). Chemiluminescence Evaluation of Antioxidant Activity and Prevention of DNA Damage Effect of Peptides Isolated from Soluble Eggshell Membrane Protein Hydrolysate. J. Agric. Food Chem..

[17] Shi Y., Kovacs-Nolan J., Jiang B., Tsao R., Mine Y. (2014). Peptides derived from eggshell membrane improve antioxidant enzyme activity and glutathione synthesis against oxidative damage in Caco-2 cells. J. Funct. Foods.

[18] Glatz P., Miao Z., Rodda B. (2011). Handling and Treatment of Poultry Hatchery Waste: A Review. Sustainability.

[19] King’Ori A.M. (2011). A Review of the uses of poultry eggshells and shell membranes. Int. J. Poult. Sci..

[20] Saratale R.G., Sun Q., Munagapati V.S., Saratale G.D., Park J., Kim D.S. (2021). The use of eggshell membrane for the treatment of dye-containing wastewater: Batch, kinetics and reusability studies. Chemosphere.

[21] Cordeiro C.M.M., Hincke M.T. (2016). Quantitative proteomics analysis of eggshell membrane proteins during chick embryonic development. J. Proteom..

[22] Rath N.C., Liyanage R., Makkar S.K., Lay J.O. (2017). Protein profiles of hatchery egg shell membrane. Proteome Sci..

[23] Ahmed T.A.E., Suso H.P., Hincke M.T. (2017). In-depth comparative analysis of the chicken eggshell membrane proteome. J. Proteom..

[24] Zhu L., Xiong H., Huang X., Guyonnet V., Ma M., Chen X., Zheng Y., Wang L., Hu G. (2022). Identification and molecular mechanisms of novel antioxidant peptides from two sources of eggshell membrane hydrolysates showing cytoprotection against oxidative stress: A combined in silico and in vitro study. Food Res. Int..

[25] Sykiotis G.P. (2021). Keap1/Nrf2 Signaling Pathway. Antioxidants.

[26] AOAC (2005). Official Methods of Analysis of the Association of Official Analytical Chemists.

[27] Jia Y., Wang Y., Li R., Li S., Zhang M., He C., Chen H. (2021). The structural characteristic of acidic-hydrolyzed corn silk polysaccharides and its protection on the HO-injured intestinal epithelial cells. Food Chem..

[28] Liu D., Guo Y., Wu P., Wang Y., Kwaku Golly M., Ma H. (2020). The necessity of walnut proteolysis based on evaluation after in vitro simulated digestion: ACE inhibition and DPPH radical-scavenging activities. Food Chem..

[29] Zhang Q., Tong X., Li Y., Wang H., Wang Z., Qi B., Sui X., Jiang L. (2019). Purification and Characterization of Antioxidant Peptides from Alcalase-Hydrolyzed Soybean (*Glycine max* L.) Hydrolysate and Their Cytoprotective Effects in Human Intestinal Caco-2 Cells. J. Agric. Food Chem..

[30] Kang B., Skonberg D.I., Myracle A.D. (2020). Anti-Hyperglycemic Effects of Green Crab Hydrolysates Derived by Commercially Available Enzymes. Foods.

[31] Wang L., Yang M., Zhang C., Huang F. (2020). The protective effects of dehydrocostus lactone against TNF-α-induced degeneration of extracellular matrix (ECM) in SW1353 cells. Aging.

[32] Jain S., Anal A.K. (2016). Optimization of extraction of functional protein hydrolysates from chicken egg shell membrane (ESM) by ultrasonic assisted extraction (UAE) and enzymatic hydrolysis. LWT-Food Sci. Technol..

[33] Wang Y., Wang Z., Handa C.L., Xu J. (2017). Effects of ultrasound pre-treatment on the structure of β-conglycinin and glycinin and the antioxidant activity of their hydrolysates. Food Chem..

[34] Park S., Choi K.S., Lee D., Kim D., Lim K.T., Lee K.-H., Seonwoo H., Kim J. (2016). Eggshell membrane: Review and impact on engineering. Biosyst. Eng..

[35] Shi Y., Zhou K., Li D., Guyonnet V., Hincke M.T., Mine Y. (2021). Avian Eggshell Membrane as a Novel Biomaterial: A Review. Foods.

[36] Rose-Martel M., Smiley S., Hincke M.T. (2015). Novel identification of matrix proteins involved in calcitic biomineralization. J. Proteom..

[37] Li Y., Li Y., Liu S., Tang Y., Mo B., Liao H. (2018). New zonal structure and transition of the membrane to mammillae in the eggshell of chicken Gallus domesticus. J. Struct. Biol..

[38] Chien Y.C., Hincke M.T., Vali H., McKee M.D. (2008). Ultrastructural matrix-mineral relationships in avian eggshell, and effects of osteopontin on calcite growth in vitro. J. Struct. Biol..

[39] Lomholt J.P. (1976). The development of the oxygen permeability of the avian egg shell and its membranes during incubation. J. Exp. Zool..

[40] Rodriguez-Navarro A.B., Marie P., Nys Y., Hincke M.T., Gautron J. (2015). Amorphous calcium carbonate controls avian eggshell mineralization: A new paradigm for understanding rapid eggshell calcification. J. Struct. Biol..

[41] Ahmed T.A.E., Suso H.P., Maqbool A., Hincke M.T. (2019). Processed eggshell membrane powder: Bioinspiration for an innovative wound healing product. Mater. Sci. Eng. C.

[42] Wen C., Zhang J., Feng Y., Duan Y., Ma H., Zhang H. (2020). Purification and identification of novel antioxidant peptides from watermelon seed protein hydrolysates and their cytoprotective effects on H_2_O_2_-induced oxidative stress. Food Chem..

[43] Liu C.M., Wang F., Zhong J.Z., Yang X., Ru-Yan D., Zhong Y.J. (2016). Functional properties and amino acid composition of cashew nut protein. Sci. Technol. Food Ind..

[44] Kaweewong K., Garnjanagoonchorn W., Jirapakkul W., Roytrakul S. (2013). Solubilization and identification of hen eggshell membrane proteins during different times of chicken embryo development using the proteomic approach. Protein J..

[45] Wong F.C., Xiao J.B., Wang S.Y., Ee K.Y., Chai T.T. (2020). Advances on the antioxidant peptides from edible plant sources. Trends Food Sci. Technol..

[46] Zamorano-Apodaca J.C., Garcia-Sifuentes C.O., Carvajal-Millan E., Vallejo-Galland B., Scheuren-Acevedo S.M., Lugo-Sanchez M.E. (2020). Biological and functional properties of peptide fractions obtained from collagen hydrolysate derived from mixed by-products of different fish species. Food Chem..

[47] Mune M.A., Minka S.R., Henle T. (2018). Investigation on antioxidant, angiotensin converting enzyme and dipeptidyl peptidase IV inhibitory activity of Bambara bean protein hydrolysates. Food Chem..

[48] Piotrowicz I.B.B., Garces-Rimon M., Moreno-Fernandez S., Aleixandre A., Salas-Mellado M., Miguel-Castro M. (2020). Antioxidant, Angiotensin-Converting Enzyme Inhibitory Properties and Blood-Pressure-Lowering Effect of Rice Bran Protein Hydrolysates. Foods.

[49] Habinshuti I., Mu T.H., Zhang M. (2020). Ultrasound microwave-assisted enzymatic production and characterisation of antioxidant peptides from sweet potato protein. Ultrason Sonochem..

[50] Zhang F., Qu J., Thakur K., Zhang J.G., Mocan A., Wei Z.J. (2019). Purification and identification of an antioxidative peptide from peony (*Paeonia suffruticosa* Andr.) seed dreg. Food Chem..

[51] Zhang Y., Dong Y., Dai Z. (2021). Antioxidant and Cryoprotective Effects of Bone Hydrolysates from Bighead Carp (*Aristichthys nobilis*) in Freeze-Thawed Fish Fillets. Foods.

[52] Shi Y., Kovacs-Nolan J., Jiang B., Tsao R., Mine Y. (2014). Antioxidant activity of enzymatic hydrolysates from eggshell membrane proteins and its protective capacity in human intestinal epithelial Caco-2 cells. J. Funct. Foods.

[53] Sierra L., Fan H., Zapata J., Wu J. (2021). Antioxidant peptides derived from hydrolysates of red tilapia (*Oreochromis* sp.) scale. LWT.

[54] Wattanasiritham L., Theerakulkait C., Wickramasekara S., Maier C., Stevens J. (2016). Isolation and identification of antioxidant peptides from enzymatically hydrolyzed rice bran protein. Food Chem..

[55] Xu S., Shen Y., Li Y. (2019). Antioxidant Activities of Sorghum Kafirin Alcalase Hydrolysates and Membrane/Gel Filtrated Fractions. Antioxidants.

[56] Carrasco-Castilla J., Hernandez-Alvarez A.J., Jimenez-Martinez C., Jacinto-Hernandez C., Alaiz M., Giron-Calle J., Vioque J., Davila-Ortiz G. (2012). Antioxidant and metal chelating activities of peptide fractions from phaseolin and bean protein hydrolysates. Food Chem..

[57] Falade E.O., Mu T.-H., Zhang M. (2021). Improvement of ultrasound microwave-assisted enzymatic production and high hydrostatic pressure on emulsifying, rheological and interfacial characteristics of sweet potato protein hydrolysates. Food Hydrocolloid.

[58] Yang J., Huang J., Dong X., Zhang Y., Zhou X., Huang M. (2020). Purification and identification of antioxidant peptides from duck plasma proteins. Food Chem..

[59] Pang K.L., Chow Y.Y., Leong L.M., Law J.X., Ghafar N.A., Soelaiman I.N., Chin K.Y. (2021). Establishing SW1353 Chondrocytes as a Cellular Model of Chondrolysis. Life.

[60] Kim S., Na J.Y., Song K.B., Choi D.S., Kim J.H., Kwon Y.B., Kwon J. (2012). Protective Effect of Ginsenoside Rb1 on Hydrogen Peroxide-induced Oxidative Stress in Rat Articular Chondrocytes. J. Ginseng Res..

[61] Singh Y.P., Moses J.C., Bhardwaj N., Mandal B.B. (2021). Overcoming the Dependence on Animal Models for Osteoarthritis Therapeutics—The Promises and Prospects of In Vitro Models. Adv. Healthc. Mater..

[62] Zhou T.Y., Xiang X.W., Du M., Zhang L.F., Cheng N.X., Liu X.L., Zheng B., Wen Z.S. (2019). Protective effect of polysaccharides of sea cucumber Acaudina leucoprocta on hydrogen peroxide-induced oxidative injury in RAW264.7 cells. Int. J. Biol. Macromol..

[63] Zhou Z., Zhang L., Liu Y., Huang C., Xia W., Zhou H., Zhou Z., Zhou X. (2022). Luteolin Protects Chondrocytes from H_2_O_2_-Induced Oxidative Injury and Attenuates Osteoarthritis Progression by Activating AMPK-Nrf2 Signaling. Oxid. Med. Cell Longev..

[64] Chen D., Shen J., Zhao W., Wang T., Han L., Hamilton J.L., Im H.J. (2017). Osteoarthritis: Toward a comprehensive understanding of pathological mechanism. Bone Res..

[65] Milaras C., Lepetsos P., Dafou D., Potoupnis M., Tsiridis E. (2021). Association of Matrix Metalloproteinase (MMP) Gene Polymorphisms with Knee Osteoarthritis: A Review of the Literature. Cureus.

[66] Baek A., Jung S.H., Pyo S., Kim S.Y., Cho S.R. (2020). 3’-sialyllactose protects SW1353 chondrocytic cells from interleukin-1β-induced oxidative stress and inflammation. Front. Pharmacol..

[67] Yang J., Song X., Feng Y., Liu N., Fu Z., Wu J., Li T., Chen H., Chen J., Chen C. (2020). Natural ingredients-derived antioxidants attenuate H_2_O_2_-induced oxidative stress and have chondroprotective effects on human osteoarthritic chondrocytes via Keap1/Nrf2 pathway. Free Radic. Biol. Med..

[68] Boyenle I.D., Divine U.C., Adeyemi R., Ayinde K.S., Olaoba O.T., Apu C., Du L., Lu Q., Yin X., Adelusi T.I. (2021). Direct Keap1-kelch inhibitors as potential drug candidates for oxidative stress-orchestrated diseases: A review on In silico perspective. Pharmacol. Res..

[69] Chen X., Li Z., Hong H., Wang N., Chen J., Lu S., Zhang H., Zhang X., Bei C. (2021). Xanthohumol suppresses inflammation in chondrocytes and ameliorates osteoarthritis in mice. Biomed. Pharmacother..

[70] Ahmed S.M., Luo L., Namani A., Wang X.J., Tang X. (2017). Nrf2 signaling pathway: Pivotal roles in inflammation. Biochim. Biophys. Acta Mol. Basis Dis..

