# Supplementary material for: Hatched Eggshell Membrane Can Be a Novel Source of Antioxidant Hydrolysates to Protect against H2O2-Induced Oxidative Stress in Human Chondrocytes
Source: Antioxidants (Basel). 2022 Dec 9;11(12):2428. doi: 10.3390/antiox11122428 (PMC9774709; doi:10.3390/antiox11122428)
Supplement: Supplementary file 1 [file antioxidants-11-02428-s001.zip › antioxidants-1948995-supplementary.pdf]

Supplementary Material

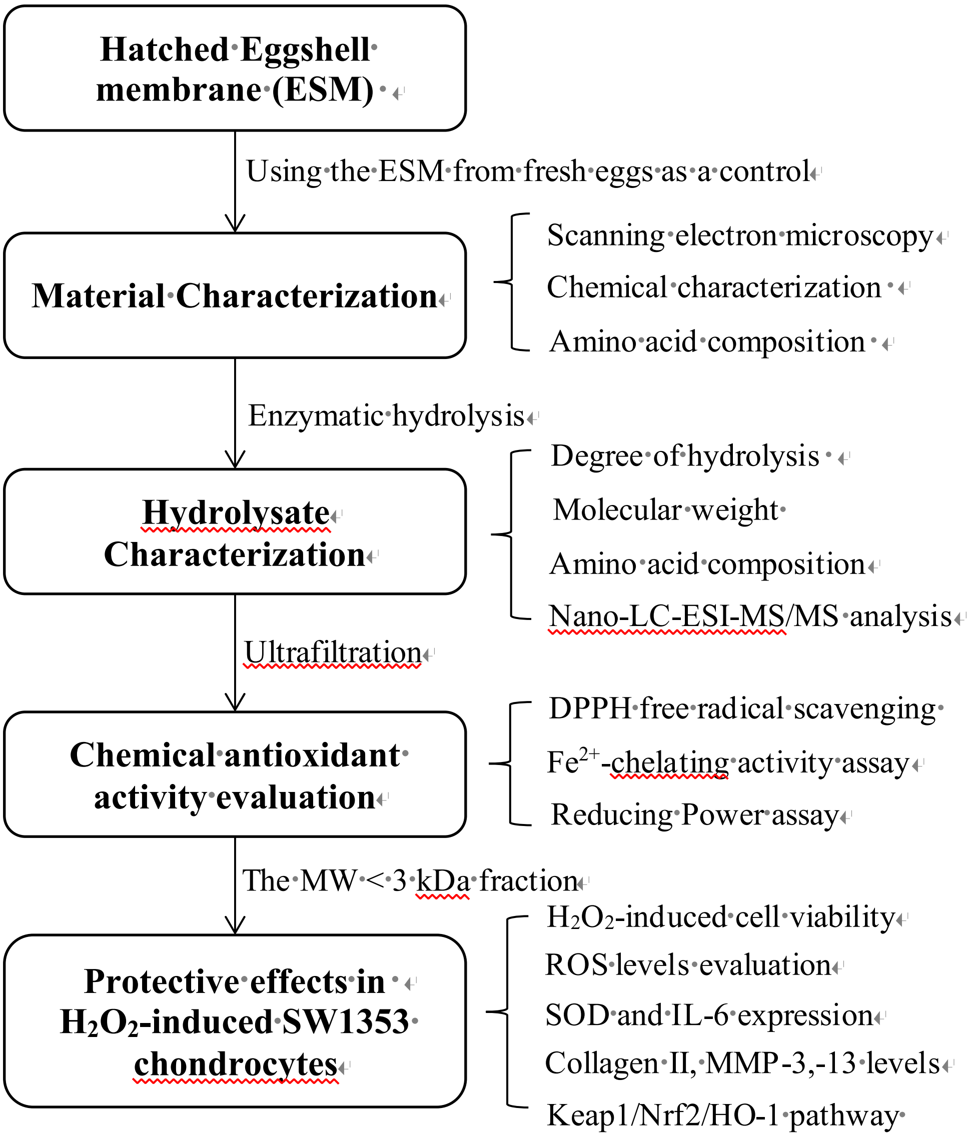

Figure S1 The flow chart of the research strategy of the present work.

A

B

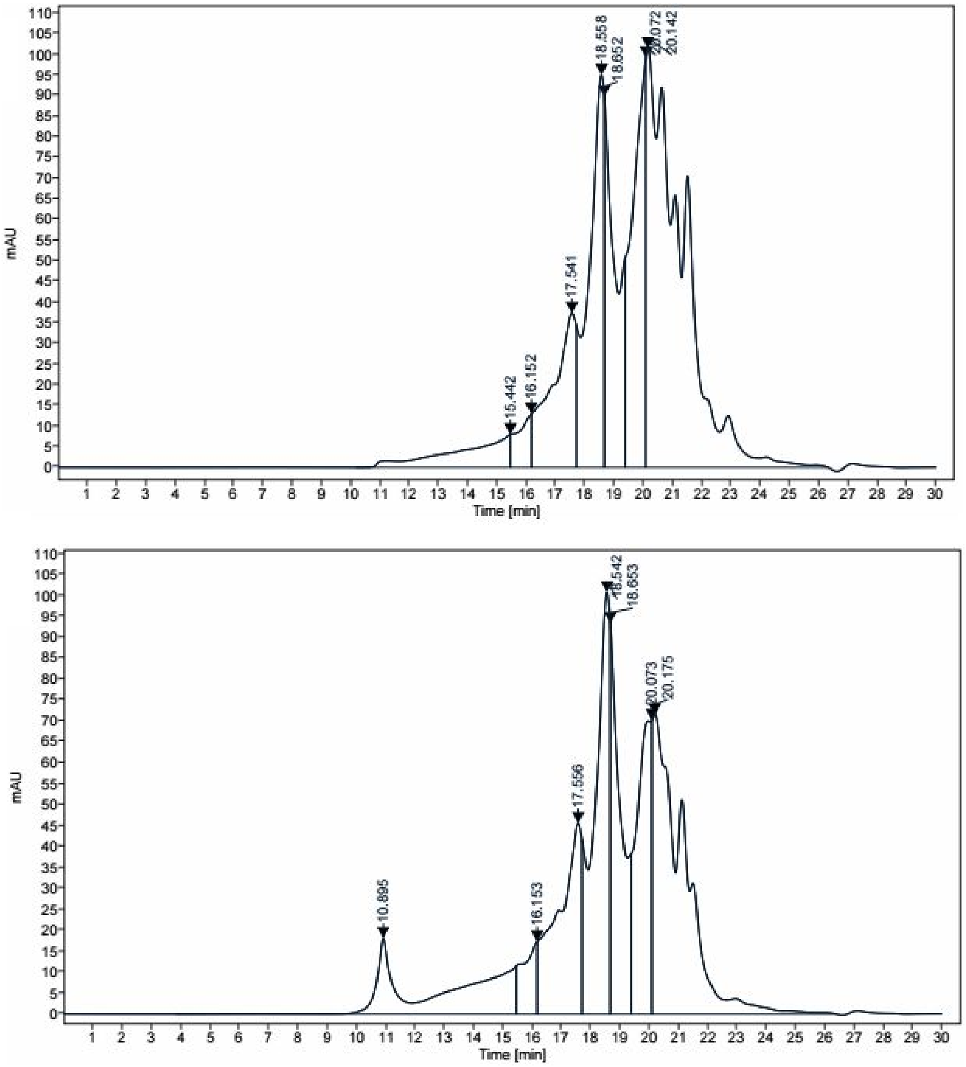

Figure S2. Molecular weight distribution of hatched eggshell membrane hydrolysates (A) and fresh eggshell membrane hydrolysates (B) by HPLC using a TSK gel G2000 column.

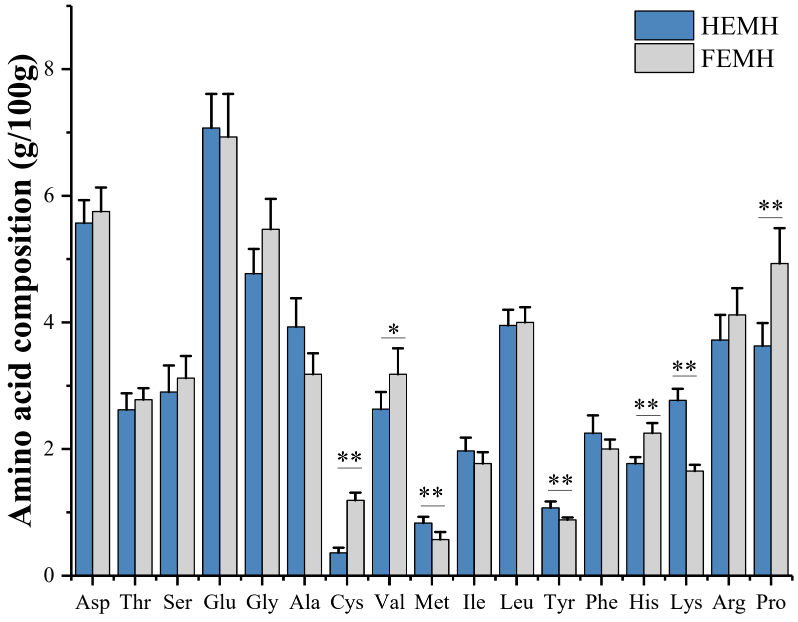

Figure S3. The composition of amino acids (g/100g) in hydrolysates of hatched ESM (HEMH) and fresh ESM (FEMH). The significance levels of *$P < 0.05$ and **$P < 0.01$ are for FEMH versus HEMH at the same amino acid.

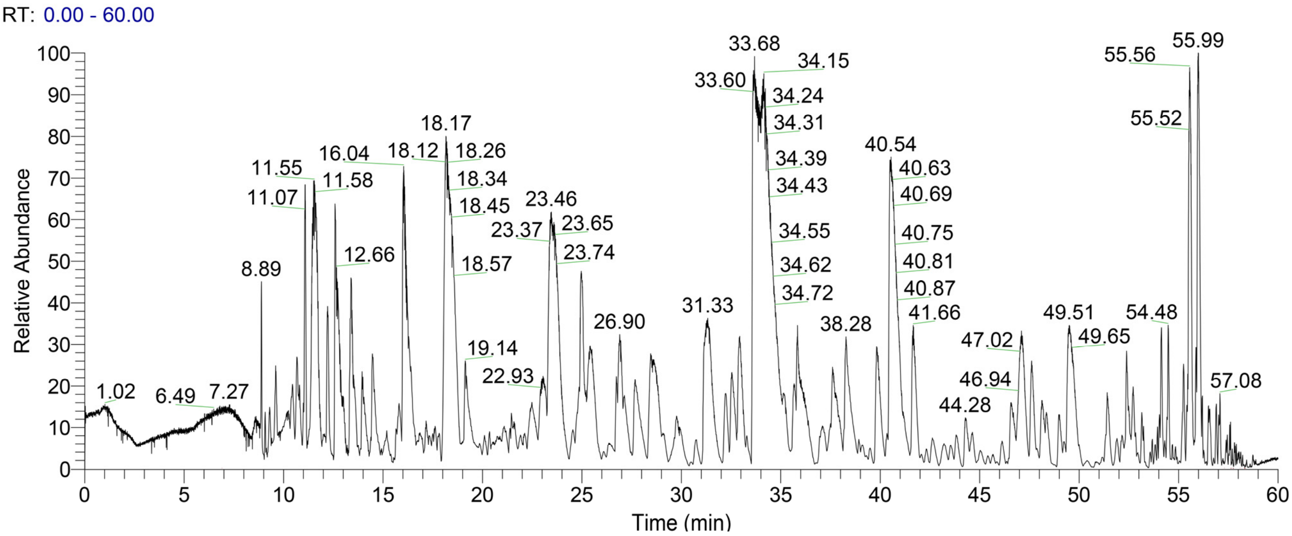

Figure S4. Total ion chromatogram by Nano-LC-ESI-MS/MS.

Table S1. The major peptides identified in HEMH by Nano-LC-ESI-MS/MS.

Sequence	Mass	Protein source	Score	Intensity (% of area)	Peptide Ranker score
VEDIRIRPIL	1222.7398	Lysyl oxidase homolog	119.29	7.70×10^9(8.58%)	0.29
NDGRTPGSR	958.45806	Lysozyme C	98.048	5.62×10^9(6.27%)	0.30
DVPRDPWVW	1168.5665	Ovocleidin-116	69.423	5.52×10^9(6.15%)	0.84
VGIGKPGPM	854.46841	Collagen alpha-1(X) chain	73.039	4.84×10^9(5.40%)	0.56
VERLRIPL	994.62875	Uncharacterized protein	110.61	4.24×10^9(4.72%)	0.22
VEDIRIRPI	1109.6557	Lysyl oxidase homolog	101.28	3.87×10^9(4.31%)	0.29
AYLEDRPMFM	1271.5679	Lysyl oxidase homolog	135.83	3.73×10^9(4.16%)	0.77
VFFDMFNAL	1102.5158	Calcium-transporting ATPase	85.265	3.71×10^9(4.14%)	0.83
DKDGPFRL	946.48723	VWFD domain-containing protein	130.22	3.15×10^9(3.51%)	0.84
AYLEDRPMFML	1384.6519	Lysyl oxidase homolog	126.07	3.0×10^9(3.34%)	0.82
